# Non-invasive capnodynamic mixed venous oxygen saturation during major changes in oxygen delivery

**DOI:** 10.1007/s10877-021-00762-5

**Published:** 2021-10-05

**Authors:** Anders Svedmyr, Mark Konrad, Mats Wallin, Magnus Hallbäck, Per-Arne Lönnqvist, Jacob Karlsson

**Affiliations:** 1 Karolinska Institute Department of Physiology and Pharmacology (FYFA), C3, Per-Arne Lönnqvist Group - Section of Anesthesiology and Intensive Care, Anestesi- och Intensivvårdsavdelningen, 171 76 Stockholm, PA Sweden; 2 Pediatric Perioperative Medicine and Intensive Care, Karolinska University Hospital, Eugenivägen 23, 171 64 Stockholm, Sweden; 3 Maquet Critical Care AB, Röntgenvägen 2, 171 06 Solna, Sweden

**Keywords:** Hemodynamic monitoring, Carbon dioxide, Cardiac output, Pulmonary artery, Oxygen, Oximetry

## Abstract

Mixed venous oxygen saturation (SvO_2_) is an important variable in anesthesia and intensive care but currently requires pulmonary artery catheterization. Recently, non-invasive determination of SvO_2_ (Capno-SvO_2_) using capnodynamics has shown good agreement against CO-oximetry in an animal model of modest hemodynamic changes. The purpose of the current study was to validate Capno-SvO_2_ against CO-oximetry during major alterations in oxygen delivery. Furthermore, evaluating fiberoptic SvO_2_ for its response to the same challenges. Eleven mechanically ventilated pigs were exposed to oxygen delivery changes: increased inhaled oxygen concentration, hemorrhage, crystalloid and blood transfusion, preload reduction and dobutamine infusion. Capno-SvO_2_ and fiberoptic SvO_2_ recordings were made in parallel with CO-oximetry. Respiratory quotient, needed for capnodynamic SvO_2_, was measured by analysis of mixed expired gases. Agreement of absolute values between CO-oximetry and Capno-SvO_2_ and fiberoptic SvO_2_ respectively, was assessed using Bland–Altman plots. Ability of Capno- SvO_2_ and fiberoptic SvO_2_ to detect change compared to CO-oximetry was assessed using concordance analysis. The interventions caused significant hemodynamic variations. Bias between Capno-SvO_2_ and CO-oximetry was + 3% points (95% limits of agreements – 7 to + 13). Bias between fiberoptic SvO_2_ and CO-oximetry was + 1% point, (95% limits of agreements − 7 to + 9). Concordance rate for Capno-SvO_2_ and fiberoptic SvO_2_ vs. CO-oximetry was 98% and 93%, respectively. Capno-SvO_2_ generates absolute values close to CO-oximetry. The performance of Capno-SvO_2_ vs. CO-oximetry was comparable to the performance of fiberoptic SvO_2_ vs. CO-oximetry. Capno-SvO_2_ appears to be a promising tool for non-invasive SvO_2_ monitoring.

**Supplementary Information:**

The online version contains supplementary material available at 10.1007/s10877-021-00762-5.

## Introduction

Mixed venous oxygen saturation (SvO_2_) is a clinically useful monitoring parameter that provide important information regarding the whole-body oxygen delivery/consumption balance and is of particular interest in the setting of anaesthesia and intensive care [1]. The use of SvO_2_ as a routine monitoring parameter has so far been hampered by the fact that intermittent blood sampling from the pulmonary artery (CO-oximetry; representing “gold standard”) as well as continuous assessment by fibreoptic spectrophotometry both require invasive catherization of the pulmonary artery, which is associated with certain risks as well as increased nursing care demands and cost [2, 3].

During our development of a modern version of capnodynamic assessment of cardiac output (Effective Pulmonary Blood Flow, CO_EPBF_), which we have found to produce similar results when compared to accepted reference methods, we have come to realize that combining CO_EPBF_ with the respiratory quotient (RQ) results in the possibility to determine SvO_2_ noninvasively [4–7]. In a recent proof-of-concept study we have found that this new non-invasive and continuous methodology of monitoring SvO_2_ (Capno-SvO_2_) show promising results when compared to CO-oximetry determinations of SvO_2_ in a porcine model, during modest hemodynamic challenges [8].

The aim of the present study was to validate Capno-SvO_2_ against the gold standard CO-oximetry for agreement of absolute values and ability to detect change during profound variations in oxygen delivery while measuring RQ, using our previously well-established porcine model. A comparison between CO-oximetry and another continuous SvO_2_ methodology, i.e., fibreoptic SvO_2,_ was also included for comparison.

## Materials and methods

### Animal preparations

The study was performed at the Hedenstierna Laboratory, Uppsala University, Uppsala Sweden. Authorization was granted from Uppsala Animal Ethics Committee (Uppsala, Sweden case number C75/16, chairperson Erik Göransson on August 26, 2016).

The animals were handled in accordance with the animal experimentation guidelines of the Uppsala Animal Ethics Committee and Animal Research: Reporting of In Vivo Experiments (ARRIVE) guidelines.

Eleven domestic-breed pigs of both sexes (median weight 29.6 kg, range 27.1–31.7 kg, 6–8 weeks of age), from the same breeding colony (Mångsbo Farm, Uppsala, Sweden) were used. The animals were kept in a light- and temperature-controlled environment, with unlimited access to tap water and food on a standardized schedule. The experiments were done during daytime hours.

The pigs were anaesthetized as previously described and mechanically ventilated in a volume-controlled mode (Servo-I; Maquet, Solna, Sweden), with tidal volume of 10 mL kg^−1^ and fraction of inspired oxygen (FiO_2_) 0.3 [9]. Positive end-expiratory pressure (PEEP) was kept at 5 cm H_2_O after an initial 2-min period of lung expansion using PEEP 10 cm H_2_O as previously described [8]. After the lung expansion manoeuvre, an air test using FiO_2_ 0.21 was performed and repeated if necessary, aiming for sustained pulse oximetry saturation > 97% as indicative of open lung conditions [10]. The animals were given a bolus of Ringers’ acetate solution 20 mL kg^− 1^ after induction and thereafter kept on maintenance infusion of glucose 25 mg mL ^−1^ 8 mL kg^−1^ h^−1^ and Ringer’s acetate solution 10 mL kg^−1^ h^−1^. Exhaled CO_2_ was measured by a mainstream infrared CO_2_ sensor (Capnostat-3; Respironics Inc, Wallingford, CT), and ventilation airflow was registered through the regular flow sensor of the servo-I ventilator. Volumetric capnography was utilised for calculating the CO_2_ elimination rate (VCO_2_), as described in previous studies [11].

Adequate anesthetic depth and analgesic level were tested regularly during the experiment according to standard procedures of the laboratory. The animals were fitted with monitoring devices described in detail in previous studies [9]. In addition a 7.5 F pulmonary artery catheter with fibreoptic SvO_2_ monitoring (Swan-Gantz pulmonary artery catheter, model 774F75; Edwards Lifesciences, Irvine CA; USA) and a 5 F femoral artery cannula for transpulmonary thermodilution cardiac output monitoring (PICCO2™, Pulsion Medical Systems, Munich, Germany), were also included as well as an additional 7.5 F pulmonary artery introducer sheath for rapid fluid administration.

A 12 F balloon tipped catheter (Mediq Sverige AB, Uppsala, Sweden) was surgically introduced through the left femoral vein, to a depth of 30 cm corresponding to the inferior vena cava (IVC). The catheter was fitted with a 10 mL balloon allowing partial IVC occlusion when inflated with NaCl 0.9%. The animals were given a bolus dose of intravenous heparin 5000 U (LEO pharma) to minimize the risk of clotting due to the extensive intravascular monitoring setup.

SvO_2_ through blood samples were analysed by a CO-oximeter calibrated for porcine hemoglobin (OSM3; Radiometer Medical AbS, Brønshøj, Denmark). This also provided the hemoglobin level, necessary for the calculations of CO_EPBF_ and Capno-SvO_2_ as well as for calibrations of fiberoptic SvO_2_.

### Assessment of Capno-SvO_2_

The principle behind continuous Capno-SvO_2_ method has been described in detail in previous work by our research team [8]. The Capno-SvO_2_ method is based on the differential Fick principle and utilizes a combination of continuous estimation of CO_EPBF_ and oxygen consumption (VO_2_) incorporated in a rearranged Fick’s equation [12]. CO_EPBF_ is estimated by applying a special breathing pattern of variations in I:E relationships (six breaths with normal I:E relations followed by three breaths vid an approximately 2 s expiratory pause). This breathing pattern causes small fluctuations in alveolar CO_2_ concentration and VCO_2_, related to the pulmonary blood flow participating in gas exchange which allows for estimation of CO_EPBF_ [5, 7, 13]. VO_2_ can be estimated using continuous volumetric capnography measurement of VCO_2_ combined with RQ:

1 $$V{O}_{2}=\frac{VC{O}_{2}}{RQ}$$

where VO_2_ is the oxygen consumption (mL min^− 1^), VCO_2_ is the CO_2_ production rate (mL min^− 1^), RQ is the respiratory quotient.

VCO_2_ applied in Eq. 1 is determined as a moving mean value of measured CO_2_ elimination made over a period of 20 min in order to produce stable VCO_2_ values with the intent to reflect an assumed stable metabolism.

Using Fick’s equation for oxygen, the content of oxygen in mixed venous blood (CvO_2_) can be derived:

2 $$C{O}_{EPBF}=\frac{V{O}_{2}}{Cc{O}_{2}-Cv{O}_{2}}$$

where CO_EPBF_ is the effective pulmonary blood flow (L min^− 1^), VO_2_ is the oxygen consumption (mL min^− 1^), CcO_2_ is the pulmonary end capillary oxygen content (mL L^− 1^), CvO_2_ is the pulmonary mixed venous oxygen content (mL L^− 1^).

Equation 2 can be further rearranged to derive CvO_2_:

3 $$Cv{O}_{2}=Cc{O}_{2}-\frac{V{O}_{2}}{C{O}_{EPBF}}$$

where CO_EPBF_ is the effective pulmonary blood flow (L min^− 1^), VO_2_ is the oxygen consumption (mL min^− 1^), CcO_2_ is the pulmonary end capillary oxygen content (mL L^− 1^), CvO_2_ is the pulmonary mixed venous oxygen content (mL L^− 1^).

CvO_2_ from Eq. 3 can then be used to calculated SvO_2_ by using the solubility constant for oxygen in blood plasma, Hüfner’s constant and the hemoglobin value as previously described thereby generating breath-by-breath estimations of SvO_2_ [8].

### RQ-measurements

**Fig. 1:**
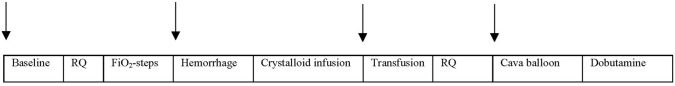
Scheme of the study protocol. *RQ* respiratory quotient; (FiO_2_), FiO_2_ steps. Black arrows indicate calibration points were fiberoptic SvO_2_ was calibrated against CO-oximetry and hemoglobin and Capno-SvO_2_ against current hemoglobin

The RQ was determined immediately before the start of the first challenge (morning) and immediately before the start of the last challenge (afternoon), approximately 4 h apart (shown in Fig. 1). Measurements were performed in all animals, by analysis of mixed expired gas collected in a Douglas bag. Each RQ measurement was conducted in the following way:

- Ventilator bias flow during expiration phase ((normally 2 L min^−1^ in Servo-I ventilator (Maquet, Solna, Sweden)) was turned off to avoid diluting the gas in the bag with inspiration gas.
- FiO_2_ was changed from 0.30 to 0.21 (i.e., room air), which was done for two reasons; it gives the best conditions for the RQ calculation (as shown in Eq. 1 below) and the inspired gas concentration is well-defined without relying on accurate gas mixing by the ventilator.
- To reach a stable state of nitrogen (N_2_), 20 min wash-in of N_2_ was allowed after the change of FiO_2_ and before the bag was connected to the ventilator exhaust port.
- Gas was collected for about 10 min; roughly 70 L of expiratory gas was collected.
- The content of the bag was analyzed using the side-stream gas analyzer of a Flow-i anesthesia system (Maquet, Solna, Sweden). Gas was alternatingly sampled from the bag or from the room air.

Gas concentrations of FiO_2_ (room air), FemixO_2_ (bag) and FemixCO_2_ (bag) were measured at ATPD state (Ambient temperature and pressure, dry). RQ was calculated using the following formula, which is based on the Haldane transformation to account for the imbalance between inspiration and expiration volumes:

$$RQ=\frac{\left(1-Fi{O}_{2}\right)\cdot FemixC{O}_{2}}{\left(1-FemixC{O}_{2}\right)\cdot Fi{O}_{2}-Femix{O}_{2}}$$

RQ is the respiratory quotient, FiO_2_ is the fraction of inspired oxygen, FemixCO_2_ is the carbon dioxide concentration in the Douglas bag, FemixO_2_ is the oxygen concentration in the Douglas bag.

A detailed derivation of the RQ-equation can be found in Supplemental Description Content 1.

### Study protocol

After preparation, the animals were allowed a 20-minute stabilizing period. Following this, an arterial blood sample was taken for calibration of the fiberoptic (Hb and CO-oximetry SvO_2_ as per the manufacturers recommendation) and the capnodynamic monitoring system (Hb). Subsequently, five paired baseline measurement of all three methods were performed, 1 min apart, for calculation of inherent precisions. After this, the animals were subjected to six different hemodynamic challenges anticipated to affect SvO_2_ (Fig. 1):

1. Stepwise increase in FiO_2_: (0.21–0.4–0.75–1.0). Each FiO_2_ level was applied for 3 min and simultaneous SvO_2_ recordings were made at the end of each FiO_2_ level. After this, FiO_2_ was returned to baseline 0.3 and an air test was performed to ensure open lung conditions after FiO_2_ 1.0 and lungs expanded as described if needed as described above.
2. Hemorrhage: 15 mL kg^−1^ of blood were drawn under 5–10 min and kept in a plastic bag intended for donor blood, containing 5000 U of Heparin (LEO pharma). SvO_2_ values were recorded just before and 5 min after the hemorrhage.
3. Crystalloid bolus: 15 mL kg^−1^ of Ringer’s acetate solution was given centrally in the 7.5 F PA introducer sheath to simulate crystalloid resuscitation of acute hypovolemia, over 5 min, 6 simultaneous SvO_2_ measurements were obtained: before crystalloid infusion and 0, 5, 10, 15 and 20 min after completions of infusion where 0 is just after completed infusion.
4. Blood transfusion: blood drawn under step 2 was transfused back over 5–10 min and SvO_2_ data were recorded before transfusion and 0, 5, 10, 15 and 20 min after completion of the transfusion where 0 is just after completed infusion.
5. Cava balloon: the cava balloon was inflated (reducing cardiac output by 30–50%). SvO_2_ data was collected prior to inflating the balloon, 2 min after inflation, and 3 min after deflation.
6. Dobutamine: a central infusion of dobutamine (Hameln pharma gmbh, Hameln, Germany) 10 mcg kg^−1^ min^−1^ was started immediately after the 3-min recording after cava balloon deflation. SvO_2_ was then recorded 5, 10 and 15 min after starting the dobutamine infusion.

The fiberoptic SvO_2_ measurement was calibrated with Hb-level and CO-oximetry SvO_2_ values as per manufacturer’s instructions. This was performed totally 4 times during the experiment as marked in Fig. 1 to ensure continuous optimal performance during the experiment. At the same time points, the capnodynamic method was calibrated with the corresponding Hb-level. Throughout the study protocol, Capno-SvO_2_ and fiberoptic SvO_2_, were recorded just before the blood samples for CO-oximetry were drawn. Due to previous experiences from caval occlusion causing extensive hemodynamic strain on the animals, no baseline recordings were made before the dobutamine step. Instead, dobutamine was started immediately after the last recording of step 5 as described above. The 3-minute recordings after cava balloon deflation, i.e., just before start of the dobutamine infusion, was used as comparison point for concordance analysis for the dobutamine step.

The animals were euthanized at the end of the experiment, according to the laboratory’s routines.

### Statistical analysis

Raw data for absolute values of all three SvO_2_ methods (CO-oximetry, Capno-SvO_2_ and fibreoptic SvO_2_), as well as paired differences between Capno-SvO_2_ and CO-oximetry and between fibreoptic SvO_2_ and CO-oximetry, were controlled for normal distribution by D’Agostino and Pearson test and visual inspection of the corresponding histograms. Values are presented as mean and 95% confidence interval (CI).

The first five measurements, during hemodynamically stable baseline conditions, were used for calculation of inherent precision for each SvO_2_ method respectively. Inherent precision was defined as 2 times the coefficient of variation (CV = SD/mean) and was in turn used to quantify the least significant change (LSC) for the reference method CO-oximetry [14]. The LSC reflects the minimum change measured and recognized as a true change [14]. LSC was then used to determine the exclusion zone in the concordance analysis as previously described [8].

Bland–Altman analysis corrected for repeated measurements was used to assess agreement of absolute values between paired recording of CO-oximetry and Capno-SvO_2_ as well as between CO-oximetry and fibreoptic SvO_2_. Bias was defined as the mean difference between the tested methods and the reference method [15, 16]. Limits of agreement (bias ± [1.96 × SD]) were used when presenting the spread of included data points and are shown with the corresponding 95% CI. Mean percentage error (PE) was calculated as 1.96 × SD of the differences, divided by the mean of the reference method [17]. A predefined PE of < 30% was defined a priori to indicate a clinically useful agreement. The predefined maximum allowed difference for agreement between the tested methods and the reference method was set to 15% points (based on previous published performance of fibreoptic SvO_2_ devices and Capno-SvO_2_) [8, 18–20]. Based on our previous study, given the maximally allowed difference between the tested method and the reference method, a minimum of approximately 250 paired data points (corresponding to 10 animals with 25 paired recording per animal) was required to show that the 95% confidence interval of the limits of agreement would fall within the pre-defined maximal allowed difference thus showing that the methods are in agreement (80% power, 0.05 significance level) [8, 15, 21]. The ability of Capno-SvO_2_ and fibreoptic SvO_2_ to track changes in SvO_2_ compared to the refence method, was assessed by calculating the concordance rate, i.e., the percentage of data points moving in the same direction when comparing two different techniques [22]. Recorded changes between baseline measurements before each hemodynamic intervention, and the value corresponding to the highest change from baseline within that intervention, were used for calculation of the concordance rate. The LSC for CO-oximetry was found to be 9.5%. Based on this, changes in SVO_2_ of less than 9.5% (rounded up to 10%) of the mean of all SvO_2_ CO-oximetry recordings (corresponding to a minimal change in CO-oximetry SvO_2_ of 5% points) were not regarded as true changes and excluded from the concordance analysis [14].

Due to the increasingly unreliable performance of CO-oximetry method for low SvO_2_ levels, i.e., for values under 30%, CO-oximetry SvO_2_ values under 30% and its associated Capno-SvO_2_ and fibreoptic SvO_2_ values were excluded in the analysis [23, 24].

GraphPad Prism (version 9.0.0 for Windows, GraphPad Software, San Diego, CA, USA) and Medcalc Statistical Software version 16.8.4 (MedCalc Software, Ostend, Belgium) was used for statistical calculations and Microsoft Excel for Mac 2020 version 16.41 for data handling.

## Results

All animals survived the experiment. SvO_2_ data for step 1–3 in the protocol was excluded from one animal due malposition of the PA-catheter which was then repositioned correctly for step 4–6. The dobutamine step was introduced after the first animal and thus includes only 10 animals.

### Calculated inherent precision

The inherent precision was found to be ± 7% for CO-oximetry SvO_2_ and ± 8% and ± 6% for Capno-SvO_2_ and fiberoptic SvO_2_, respectively which is in line with previous publications [8, 19].

### RQ

The mean RQ for morning and afternoon recordings for all eleven animals, i.e., in total 22 RQ values, was found to be 0.97 (range 0.88–1.06) and RQ 0.97 was then used for all SvO_2_-capno calculations.

### Response to hemodynamic interventions

**Fig. 2:**
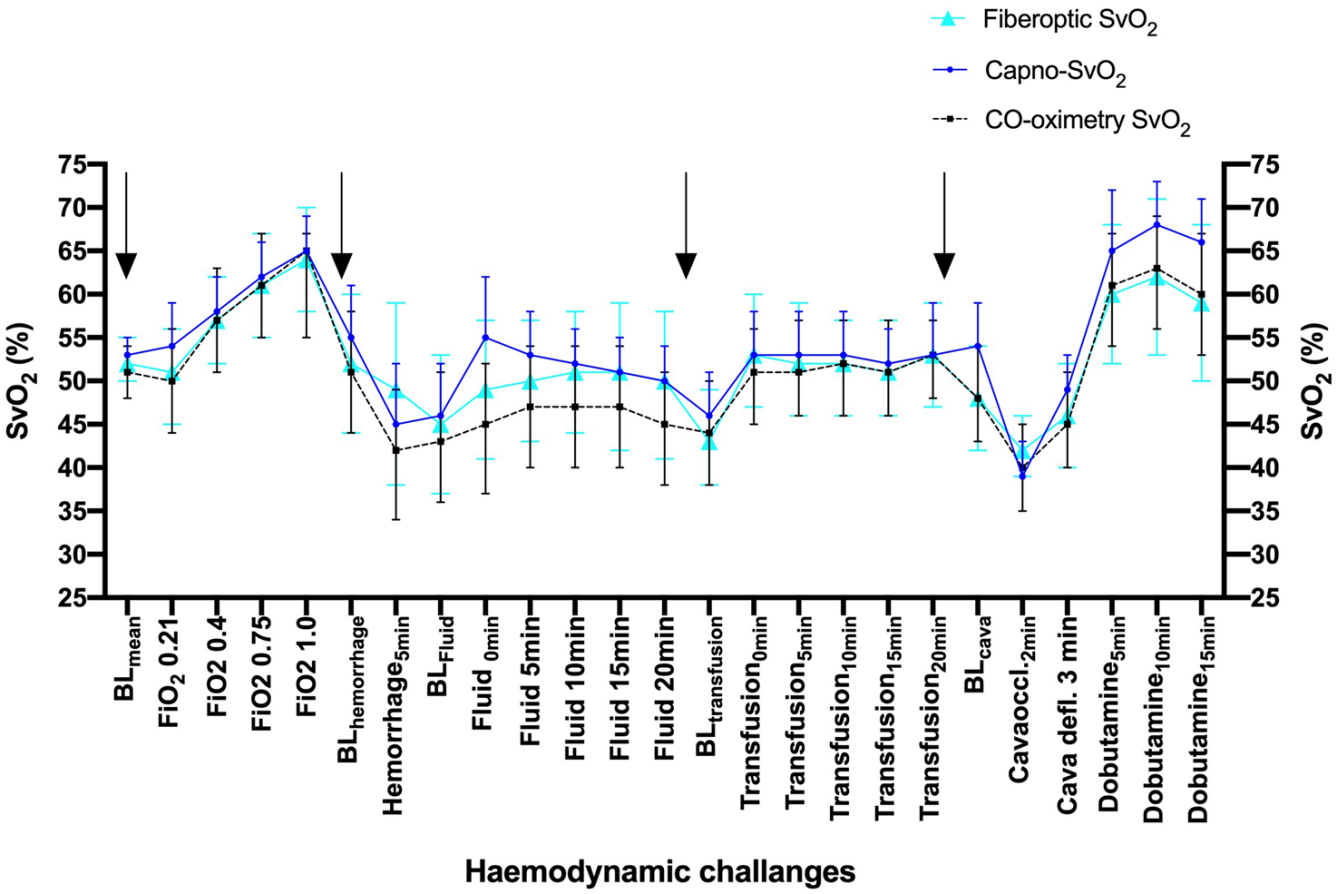
Event plot showing SvO_2_ responses to the various hemodynamic challenges for all three SvO_2_ monitoring methods. Values are mean (95% CI). N = 11. Arrows indicate recalibration points for SvO_2_-capno and fiberoptic SvO_2_. *BL* Baseline, *BL*_*mean*_ mean of precision recordings, *FiO*_*2*_ fraction inspired oxygen; *BL*_*cava*_ Baseline cava occlusion step, *Cavaoccl.2 min* 2 min after cava occlusion, *Cava defl. 3 min* 3 min after cava balloon deflation

All animals displayed SvO_2_ reactions in an anticipated manner when exposed to the various hemodynamic interventions. Both Capno-SvO_2_ and fiberoptic SvO_2_ showed parallel changes when compared to the reference method CO-oximetry with increase in SvO_2_ in response to increased FiO_2_, crystalloid infusion, blood transfusion and dobutamine administration. Correspondingly, hemorrhage and partial cava occlusion led to decreases in SvO_2_ for all methods. The changes in SvO_2_ for each hemodynamic intervention for all three SvO_2_ methods are shown in the event plot in Fig. 2.

### Agreement of absolute values

**Fig. 3:**
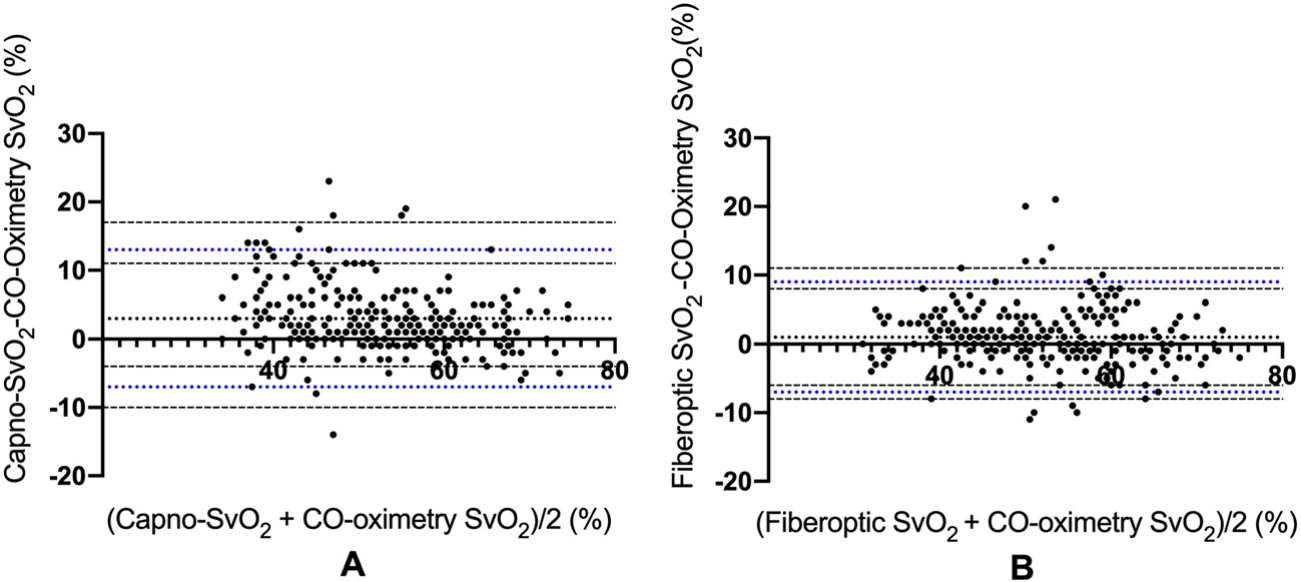
Bland–Altman plots for pooled recordings for Capno-SvO_2_ vs. CO-oximetry (**A**) and fiberoptic SvO_2_ vs. CO-oximetry (**B**). Dotted line represents bias, blue dotted lines represent upper and lower limits of agreement and black broken lines represents the corresponding CI for the limits of agreements. N = 11. **A** 271 paired data points, **B** 272 paired data points

Bland–Altman analysis for all paired data showed a bias between Capno-SvO_2_ and CO-oximetry of + 3% points with 95% limits agreement of – 7 (CI − 10 to -4) to + 13 (CI 11 to 17) percentage points and a mean percentage error of 18%. Correspondingly, the bias between fiberoptic SvO_2_ and CO-oximetry was found to be + 1% point with 95% limits of agreement of − 7 (CI − 8 to − 6) to + 9 (CI 8 to 11) percentage points and PE 16%. Bland Altman plots for Capno-SvO_2_ vs. CO-oximetry and fiberoptic SvO_2_ vs. CO-oximetry are shown in Fig. 3. Table 1 shows the agreement of absolute values between the reference method and the tested methods for each hemodynamic intervention.

**Table 1:** Bias and limits of agreement between Capno-SvO_2_ and CO-oximetry SvO_2_ as well as between fiberoptic and CO-oximetry SvO_2_ for the various hemodynamic challenges

Hemodynamic intervention	Compared methods	Bias (percentage points)	ULOA (CI) (percentage points)	LLOA(CI) (percentage points)
Baseline precision	Capno vs. CO-oxi	2	10 (7 to 16)	− 6 (− 12 to − 3)
	Fiber vs. CO-oxi	2	10 (7 to 15)	− 6 (− 12 to − 4)
FiO _2_	Capno vs. CO-oxi	1	11 (8 to 18)	− 8 (− 15 to − 5)
	Fiber vs. CO-oxi	0	7 (7 to 11)	− 7 (− 12 to − 5)
Hemorrhage	Capno vs. CO-oxi	3	14 (10 to 23)	− 8 (− 17 to − 4)
	Fiber vs. CO-oxi	2	13 (9 to 20)	− 8 (− 15 to − 4)
Crystalloid infusion	Capno vs. CO-oxi	6	17 (14 to 23)	− 6 (− 12 to − 3)
	Fiber vs. CO-oxi	3	10 (8 to 15)	− 3 (− 8 to − 1)
Transfusion	Capno vs. CO-oxi	1	7 (5 to 11)	− 5 (− 8 to − 3)
	Fiber vs. CO-oxi	1	9 (7 to 14)	− 8 (− 13 to − 6)
Cava balloon	Capno vs. CO-oxi	5	13 (10 to 19)	− 4 (− 10 to − 1)
	Fiber vs. CO-oxi	1	6 (4 to 9)	− 6 (− 8 to − 3)
Dobutamine infusion	Capno vs. CO-oxi	5	11 (8 to 17)	− 2 (− 7 to 1)
	Fiber vs. CO-oxi	− 1	8 (4 to 16)	− 10 (− 18 to − 6)

*ULOA* upper limits of agreement, *LLOA* lower limits of agreement, *CI* 95% confidence interval, *Capno* Capno-SvO_2_, *CO-oxi* CO-oximetry SvO_2_, *Fiber* fiberoptic SvO_2_

### Trending ability

**Fig. 4:**
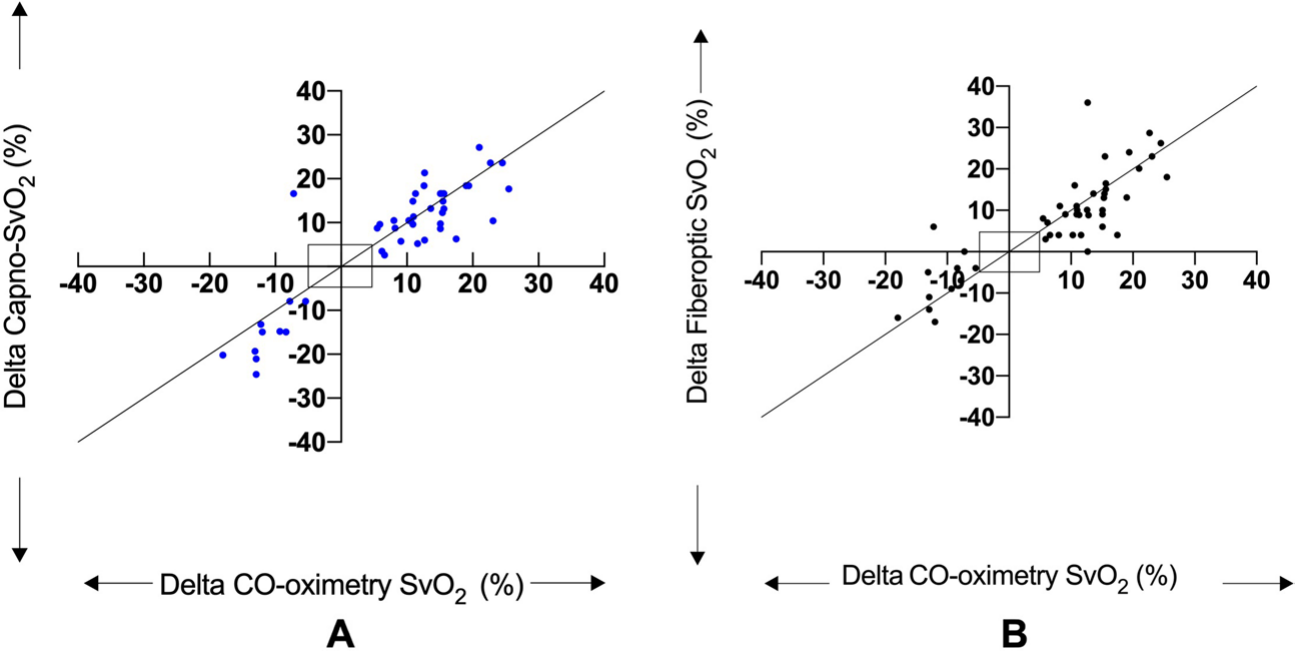
Four quadrant plots presenting the concordance between Capno-SvO_2_ and CO-oximetry SvO_2_ (**A**) and between fiberoptic SvO_2_ and CO-oximetry SvO_2_ (**B**). **A** 44 paired delta values, **B** 46 paired delta values. The central boxed area illustrates the 10% exclusion zone. Dotted line is line of identity. N = 11

The trending ability for Capno-SvO_2_ and fiberoptic SvO_2_, compared to CO-oximetry, are presented as four-quadrant plot illustrations in Fig. 4.

For Capno-SvO_2_, a total of 45 data points were found to lie outside the predefined 10% exclusion zone. 44 out of 45 values changed in the same direction as the reference method CO-oximetry which generates a concordance rate of 98%. The corresponding data for fiberoptic SvO_2_ was 46 data points outside the exclusion zone with 43 data points moving in the same direction as the reference method i.e., a concordance rate of 93%.

## Discussion

The main finding of the current study was that Capno-SvO_2_ generates absolute values close to the gold standard CO-oximetry during major hemodynamic provocations. The agreement between Capno-SvO_2_ and CO-oximetry was comparable to that found for fiberoptic SvO_2_ vs. CO-oximetry. Furthermore, Capno-SvO_2_ demonstrated a slightly more accurate ability to detect change in SvO_2_ than fiberoptic SvO_2_.

### Agreement of absolute values between CO-oximetry and the tested methods

Reliability in consistency during repeated measures and the ability to generate absolute values that corresponds acceptably with the gold standard can be considered absolute prerequisites for any new monitoring technique. During stable baseline conditions, Capno-SvO_2_ displayed an inherent precision matching those of both CO-oximetry and fiberoptic SvO_2_, thus, indicating a reliable monitoring system. During the following hemodynamic interventions Capno-SvO_2_ generated values close to the gold standard method and the overall agreement between Capno-SvO_2_ and CO-oximetry was within the predefined 95% limits of agreement of 15% points and well below the a priori determined limit for a PE of < 30%. There was an overall tendency for Capno-SvO_2_ to display slightly higher SvO_2_ values compared to CO-oximetry even if this difference was relatively small and within clinically acceptable limits. Fiberoptic SvO_2_ showed a smaller bias and narrower limits of agreement against CO-oximetry than that recorded for Capno-SvO_2_. Even if the difference in agreement against gold standard was relatively similar for both methods, the fact that fiberoptic SvO_2_ showed slightly better agreement is not entirely surprising since this value is generated from direct estimation of SvO_2_ in the actual pulmonary artery whereas the Capno-SvO_2_ is a figure derived from mathematical analysis of an induced variability exhaled CO_2_.

Both Capno-SvO_2_ and fiberoptic SvO_2_ showed variable levels of agreement against CO-oximetry depending on the type of hemodynamic intervention. During stable baseline conditions, both Capno-SvO_2_ and fiberoptic SvO_2_ showed near enough equal agreement against gold standard. This pattern was repeated for the FiO_2_ step which is reassuring since increased FiO_2_ is a common method of augmenting oxygen delivery in the clinical setting.

Differences between Capno-SvO_2_ and CO-oximetry can be attributed to mainly two factors. Firstly, issues related to CO_EPBF_ calculations i.e., disturbances in the prerequisites for the capnodynamic method, mainly the assumption of stable or slowly varying mixed venous CO_2_ content and the use of updated Hb levels. Secondly the incorporated RQ value will affect the Capno-SvO_2_ and thus its level of agreement against CO-oximetry.

The relatively high bias and limits of agreements seen during the fluid resuscitation (especially data points 0 and 5 min) and cava occlusion step is most likely due to initial disturbances in the mixed venous CO_2_ content and Hb level.

### Ability to track changes in SvO_2_ for Capno-SvO_2_ and fiberoptic SvO_2_

Agreement of absolute values is central, but in the clinical context trending ability may be even more important [25]. In this study, Capno-SvO_2_ showed a very good concordance rate when compared to the gold standard, detecting 98% of the changes, which is well in line with the established fiberoptic method. In addition, the agreement between CO-oximetry and Capno-SvO_2_ during stable baseline conditions was very good. Appropriate agreement during stable conditions in combination with a high concordance rate against CO-oximetry, makes Capno-SvO_2_ potentially interesting as an early warning sign of not only more major hemodynamic deteriorations but also minor hemodynamic changes (as shown in our previous publication) [8].

### Limitations

Even if the Capno-SvO_2_ technique showed potentially promising performance in the current study, like most monitoring methods it has inherent limitations that needs to be considered and further investigated, particularly if applied in a clinical setting.

#### Issues related to calibration of the fiberoptic method

The fiberoptic SvO_2_ method was recalibrated against CO-oximetry four times during the protocol to optimize its performance as shown in Fig. 1. Normally, the fiberoptic system is intended to be calibrated once daily and not frequently recalibrated [26]. As a consequence of these frequent recalibrations, not corresponding to normal clinical routine, the subsequent fiberoptic SvO_2_ values will have affected the performance of the fiberoptic catheter in a positive way. The Capno-SvO_2_ was recalibrated at the same time points as the fiberoptic system but only against the current Hb level since the Capno-SvO_2_ method is independent from repeated alignment against gold standard CO-oximetry obtained mixed venous saturations (as is the case for fiberoptic SvO_2_).

#### Influence of respiratory quotient

The RQ value is an important determinant in estimating Capno-SvO_2_ and will affect the agreement between Capno-SvO_2_ and CO-oximetry. Mathematically, choosing a higher RQ will elevate the Capno-SvO_2_ and vice versa. Statistically this affects accuracy, but not precision – i.e., bias will shift and will subsequently move upper and lower limit of agreements equally in parallel [14]. It will, however, not have an impact on the trending ability, maybe the most important feature of a monitoring method, as discussed above.

In this study the RQ was directly measured, and the mean value of all measurements was used for calculations of Capno-SvO_2_. In the clinical setting it is more likely that RQ will be taken from tabulated values since a direct measurement is impractical with currently available methods. Furthermore, RQ depends on fasting time and the nutritional substrate given and this needs to be taken into consideration when evaluating the recorded RQ [27, 28]. In the current study, the animals were fasted and kept on a low-level glucose maintenance infusion, a common practice during anaesthesia. The scenario may have been different if nutritional status and/or infusions had been altered regarding glucose concentration or type of substrate, something that can be of importance in the intensive care setting [27]. Despite this, using the mean RQ for the whole group still resulted in acceptable agreement and trending ability.

### Clinical applicability

In its current form, the Capno-SvO_2_ method requires controlled mechanical ventilation [7, 29]. However, most of the patients that would benefit from continuous non-invasive assessment of SvO_2_ will already be subjected to controlled mechanical ventilation both in the operating room and in the intensive care unit. Still, the fact that the method requires controlled ventilation will exclude patients that are spontaneously breathing or are treated with various modes of patient triggered ventilation.

Although Capno-SvO_2_ has the potential to considerably expand the ability to continuously monitor SvO_2_ compared to today, it is important to emphasize that Capno-SvO_2_ is not a substitute for the proper use of a pulmonary artery catheter since it does not provide information regarding relevant pressure and resistance parameters. However, it could potentially provide real time SvO_2_ monitoring in situations where a pulmonary artery catheter is neither practical or justifiable due to its invasive nature, associated risks, and cost. Thus, the potential role of Capno-SvO_2_ deserves further translational studies to define its place in monitoring of patients that are cared for in the operating room or in intensive care.

In conclusion, the current study shows that the Capno-SvO_2_ method is in close agreement with the gold standard CO-oximetry and displays good trending ability even during major changes in oxygen delivery. These features of the Capno-SvO_2_ method, in combination with its non-invasive nature, may offer a novel real time option for SvO_2_-monitoring. Even if a pulmonary artery catheter is of seminal importance for complete hemodynamic assessment, the Capno-SvO_2_ method could facilitate more advanced hemodynamic surveillance when a pulmonary artery catheter is not warranted, technically possible (such as in pediatric practice) or even contraindicated, thereby acting as a useful adjunct to more traditional hemodynamic monitoring.

## Supplementary Information

Below is the link to the electronic supplementary material.

Supplementary material 1 (PDF 45.8 kb)

## Data Availability

Not applicable.
