# Supplementary material for: Non-invasive capnodynamic mixed venous oxygen saturation during major changes in oxygen delivery
Source: J Clin Monit Comput. 2021 Oct 5;36(5):1315–24. doi: 10.1007/s10877-021-00762-5 (PMC8490846; doi:10.1007/s10877-021-00762-5)
Supplement: Supplementary file 1 — Supplementary material 1 (PDF 45.8 kb) [file 10877_2021_762_MOESM1_ESM.pdf]

Derivation of the RQ-equation

Douglas bag gas collection and analysis allow measurement of the respiratory exchange ratio (RER), which in equilibrium should be equal to the respiratory quotient (RQ). The former is related to uptake (VO_2) and elimination (VCO_2) of oxygen and carbon dioxide.

Equation 1 $$RQ = RER = \frac{VCO_2}{VO_2}$$

These in turn are determined by inspired and expired tidal volumes and mixed expired volume fractions of the gases (assuming that inspired gas is free from CO2):

Equation 2 $$VCO_2 = Ve \cdot FemixCO_2$$

Equation 3 $$VO_2 = Vi \cdot FiO_2 - Ve \cdot FemixO_2$$

The relation between inspired and expired volumes needs to be very accurately determined and to this end nitrogen (N_2, here also implicitly including Argon) may be used as an inert balance gas with zero net exchange:

Equation 4 $$VN_2 = Vi \cdot FiN_2 - Ve \cdot FemixN_2 = 0$$

Thus, based on this approach (often referred to as the Haldane transformation) inspired volume can be related to expired volume and the balance gas concentrations:

Equation 5 $$Vi = Ve \cdot \frac{FemixN_2}{FiN_2}$$

The nitrogen fractions are given by

Equation 6 $$FiN_2 = 1 - FiO_2$$

Equation 7 $$FemixN_2 = 1 - FemixO_2 - FemixCO_2$$

Substitution of all these relations into the RQ expression (Equation 1) yields after some algebraic simplifications

Equation 8 $$RQ = \frac{(1-FiO_2)\cdot FemixCO_2}{(1-FemixCO_2)\cdot FiO_2 - FemixO_2}$$

In the experiment, the gas collected in the Douglas bag also contains exhaled water vapour, not included in the equations above, that expands the volume and dilutes the concentration of the other gas substances. If the vapour somehow were to be eliminated, then the equations that are formulated for dry gases would hold. This is in fact what effectively happens when gas is sampled and dried before entering the analysis chamber of the side-stream gas analyser system. Thus, if gas concentrations are reported in a dry state (ATPD, Ambient temperature and pressure, dry) then Equation 8 can be used to calculate RQ.
